# Exosomal IL-8 derived from Lung Cancer and Colon Cancer cells induced adipocyte atrophy via NF-κB signaling pathway

**DOI:** 10.1186/s12944-022-01755-2

**Published:** 2022-12-29

**Authors:** Hairong Xiong, Jiaxin Ye, Kairu Xie, Wenjun Hu, Ning Xu, Hongmei Yang

**Affiliations:** 1 Department of Pathogenic Biology, School of Basic Medicine, Tongji Medical College, Huazhong University of Science and Technology, Wuhan, China; 2 Department of Infectious Diseases, Union Hospital, Tongji Medical College, Huazhong University of Science and Technology, Wuhan, China; 3 Wuhan National Laboratory for Optoelectronics, Huazhong University of Science and Technology, Wuhan, China; 4 Department of Clinical Laboratory, First Affiliated Hospital of Anhui Medical University, Hefei, China

**Keywords:** Cancer cachexia, Extracellular vesicles, IL-8, Lipolysis, NF-κB signaling

## Abstract

**Background:**

Cytokines secreted in the tumor microenvironment function in cancer cachexia (CC), a common clinicopathological syndrome associated with adipocyte wasting and skeletal muscle atrophy. Extracellular vesicles (EVs) secreted by cancer cells actively engage in inter-tissue communication; EVs and enclosed cytokines are largely undefined in CC adipocytes wasting.

**Methods:**

EVs derived from Lewis lung carcinoma (LLC) and colorectal cancer C26 cells were extracted and characterized. Conditioned medium and EVs from cancer cells were applied to 3 T3-L1 adipocytes. Recombinant IL-8, IL-8 neutralizing antibody, CXCR2 and NF-κB inhibitor were examined in functional assays. Lipolysis of adipocytes was monitored by Western blots, Oil red O staining and glycerol assays. Furthermore, LLC and C26 cell lines were established as cachexia model to explore the relevance of IL-8 and NF-κB signaling in CC adipose wasting. Adipose tissues were collected for histology analyses.

**Results:**

LLC and C26 cell-derived EVs induced lipolysis of 3 T3-L1 adipocytes. Specially, Dil-labeled EVs were effectively taken up by 3 T3-L1 adipocytes, which were motivated by the delivered IL-8 to elicit the NF-κB pathway. In comparison, special IL-8 neutralizing antibody relieved that lipolysis of 3 T3-L1 adipocytes induced by EVs together with conditioned medium of LLC and C26 cells, respectively. Consistently, both CXCR2 and NF-κB inhibitors would lessen the phenotype of lipolysis in 3 T3-L1 adipocytes. In the in vivo settings, both LLC and C26-tumor bearing mice had higher serum IL-8 levels as compared to the control groups. Two typical lipolysis markers, PGC1α and UCP1, were also up-regulated in the adipose tissues of LLC and C26-tumor mice groups, respectively.

**Conclusions:**

EVs secreted by LLC and C26 tumor cells would induce adipocyte wasting via extracellular IL-8-mediated NF-κB signaling. Our study pointed out the physiological and therapeutic values of exosomal IL-8 in CC lipolysis.

**Supplementary Information:**

The online version contains supplementary material available at 10.1186/s12944-022-01755-2.

## Introduction

Cancer cachexia (CC) is a multi-symptoms systemic inflammatory disease characterized by the loss of adipose and skeletal muscle weight [1]. Cachexia, caused by reduced nutrient intake and increased energy expenditure, considerably affects the quality of life. Patients with cachexia demonstrate increased proteolysis of skeletal muscles and white adipose tissue (WAT) browning [2]. Malignant tumors, such as those of the liver, lung, pancreas, stomach and intestine, are extensively associated with the development of cachexia, which causes roughly 50% of all deaths in cancer patients worldwide [3]. Cancer cachexia involves different organs and tissues, including muscle atrophy, adipose tissue depletion, and other target tissue changes. Moreover, adipose tissue loss generally precedes skeletal muscle atrophy. However, there are currently no effective strategies or approved compounds to reverse cachexia in cancer patients [4, 5]. Therefore, understanding the molecular mechanisms behind adipose tissue wasting might contribute to CC drug development.

The mechanism of CC adipose browning and lipolysis are known to be complex, involving the metabolic process in white adipose tissue that confers it brown adipose features and activates the lipolysis and thermogenesis markers PGC1α and UCP1 [6]. UCP1 is expressed in brown or beige adipocytes and functionally drives mitochondrial respiration, facilitates the conversion of electrochemical energy into heat to increase lipolysis, and induces the weight loss of adipose tissue [7]. Extracellular vesicles (EVs) vary in origins, sizes (diameter in the range of 50 to 200 nm) and transport different cargoes (e.g. mRNAs, microRNAs, proteins). Their autocrine, paracrine, and endocrine characteristics are all essential in tissue inter-communications. Known functionalities of EVs cover cell communication, immune responses, tissue recovery, epigenetic regulation, and disease progression [8–12]. For instances: EVs secreted by breast cancer cells could release miRNA-155 to promote adipocyte browning and differentiation, by remodeling metabolism in resident adipocytes through down-regulating of PPARγ [12, 13]; exosomal ciRS-133 derived from gastric carcinoma cell facilitated white adipocytes browning through targeting miRNA-133 and activating the PRDM16 pathway [13]; and EVs from Lewis lung carcinoma cells induced lipolysis of adipocytes through the IL-6/STAT3 signaling [10]. These studies provide the rationales that cargoes of EVs function in CC adipose wasting.

The physiological activities of IL-8 polymorphism were documented in inflammation and cancer, including its association with gastric, lung and pancreatic cancer cachexia [14–17]. High IL-8 expression in patients serum and cancer cells were linked to cachexia in non-small cell lung cancer and pancreatic cancer, and significantly, mice bearing with pancreatic cancer cells showed increased muscle wasting [17]. Mechanistically, IL-8 binding to a specific receptor (CXCR2) leads to receptor internalization and perturbation of multiple biological processes including inflammatory response [18]. In accordance, CXCR2 inhibition down-regulates pro-inflammatory cytokines S100a8, S100a9 and TNF-α in progressive inflammatory diseases [19]. IL-8 activated NF-κB signaling was shown to evoke cancer progression in estrogen receptor-negative breast cancer and colorectal cancer (CRC) [20, 21].

Importantly, NF-κB signaling was implicated in WAT inflammation and adipose wasting during cancer cachexia [20, 22]. In the current study, we demonstrated the induction of 3 T3-L1 adipocyte lipolysis upon co-culturing with EVs from LLC and C26 cells, partly linked with exosomal IL-8 that was derived from tumor cells. Consistently, IL-8 specific antibody mitigated NF-κB signaling activation and lipolysis in 3 T3-L1 adipocytes. Collectively, our findings uncovered that exosomal IL-8 derived from tumor cells would provoke adipose lipolysis via the NF-κB signaling, a novel cascade in connection with cancer cachexia.

## Methods

### Cultured cells

Lewis lung carcinoma (LLC) cells, colorectal cancer (C26) cells, and 3 T3-L1 pre-adipocytes cell lines were all from the American Type Culture Collection (ATCC) with short tandem repeat authentication. LLC and C26 cells were cultured in DMEM supplemented with 10% Fetal Bovine Serum (Biological Industries, CO4001, Israel) and 1% penicillin and streptomycin. 3 T3-L1 adipocytes were inoculated with DMEM with 10% New-Born Calf Serum (Every Green, 22011-8612, China) and 1% penicillin and streptomycin;

### Animals

Male C57BL/6 mice (5-6 weeks, 18-20 g, [23–25]) were purchased from Beijing HFK Bioscience (Beijing, China). The laboratory mice were randomized in three groups: control group (PBS, CN), LLC tumor bearing group (LLC-TB) and C26 tumor bearing group (C26-TB). 5 × 10^7^ LLC/C26 cells or 100 μL PBS (CN) were implanted subcutaneously under the right armpit of male mice. After day-21, inguinal white adipose tissue (iWAT), tibialis anterior (TA), epididymal white adipose tissue (eWAT), gastrocnemius (GA) muscles, heart, liver, and tumor were harvested simultaneously and weighed. Parts of above muscle and adipose tissues samples were next rapidly fixed in 4% cold paraformaldehyde. Other parts of tissues were promptly stored at − 80 °C. The mice serum was collected and centrifuged at 2000×g for 15 min at 4 °C, and stored at − 80 °C.

### Reagents

Reagents and sources: recombinant mouse CXCL15/Lungkine protein (442-LK-002, R&D Systems); anti-IL-8 (AF-208-SP, R&D Systems); CXCR2 inhibitor (SB225002, MCE); NF-κB inhibitor (BAY 11-7082, Sigma-Aldrich); Oil red O solution (Servicebio, China).

### Isolation and identification of LLC-EVs and C26 EVs

LLC and C26 cells supernatant were collected and centrifuged several times to exclude debris [10]. EVs were isolated by a Qiagen Kit (1116085) and next verified with the bicinchoninic acid (BCA) Protein Assay Kit (Thermo Fisher Scientific, USA).

### 3 T3-L1 adipocytes culture and differentiation

The cultured medium of 3 T3-L1 adipocytes containins DMEM, 10% of FBS, 0.5 mM of 1-methyl-3-isobutylxanthine (IBMX) (I5879), 1 μg/mL of insulin (I6634), 0.25 μM of dexamethasone (D4902) and 2 μM of rosiglitazone (R2408). All reagents purchased from Sigma-Aldrich. After 2 days of culture, the cells were cultivated next in DMEM with 10% of FBS and 1 μg/mL of insulin for 2 days. Adipocytes were cultured with DMEM containing 10% of FBS for 3-5 days before treatment.

Next, 3 T3-L1 adipocytes were treated with conditioned medium (50% of fresh culture medium and 50% of LLC/C26-cell-conditioned medium). For EVs and adipocytes co-culture, 0-50 μg EVs were added to the culture medium of 3 T3-L1 adipocytes for co-inoculation in 5% CO_2_ incubator at 37 °C for 24 or 48 hours, 10 μg EVs (5 μg/mL) were used in additional tests [26].

### Internalization of Dil-labeled EVs

LLC/C26 EVs were co-cultured with Dil dye (1 μM，Invitrogen) at 37 °C for 30 min and washed with 10 mL PBS, followed by centrifugation at 1000 g for 30 min [27]. The Dil-labeled EVs were next added to 3 T3-L1 adipocytes for co-culturing at 37 °C for 12 hours, and then adipocytes were washed with PBS for 3 times. Finally, adipocytes were stained with DAPI and images were acquired with Olympus FV500 fluorescence microscope (Tokyo, Japan).

### Oil red O staining

The adipocytes were fixed in 4% paraformaldehyde and washed with PBS, followed by soaking with 60% isopropanol for 20 s, staining with Oil red O staining solution (diluted it 3:2 with ultrapure water and heated at 60 °C for 30 min, followed by filtration), and then washing with 60% isopropanol and ultrapure water sequentially. Images were obtained with the Leica microscope (DMI 3000B, Germany, 200х).

### Immunofluorescence

3 T3-L1 cells were washed with PBS and immediately fixed in 4% paraformaldehyde, followed by washing with PBS, permeabilization with 0.03% Triton-X 100 for 5 min, and blocking with 5% BSA for 1 hour. Next, the cells were incubated with anti-p65NF-κB (A2547, ABclonal, China) at 4 °C for 14 hours, followed by incubation with Alexa Fluor 488-conjugated Donkey Anti-Rabbit IgG (H + L)(4412S, Cell Signaling Technology, USA) at 37 °C for 1 hour. Nuclei were stained with 10 μg/mL DAPI for 10 min. Finally, the samples were analyzed by ZEN microscope.

### Lipolysis analysis

Following the manufacturer’s instructions, levels of glycerol release were detected by kits (Jiancheng, Nanjing, China).

### Western blot

The protocol was performed as previously described. The following primary antibodies were used: anti-CXCL15 (ab197016, Abcam, 1:1000); total p65NF-κB (A2547, ABclonal, 1:1000); NF-κB p65-pS536 (3033 T, Cell Signaling Technology, 1:1000)；anti-PGC1α (A19674, ABclonal, 1:1000); anti-UCP1 (A5857, ABclonal, 1:1000); anti-α-Tubulin (66031-1-Ig, Proteintech, 1:1000); anti-LaminB1 (A11495, ABclonal, 1:1000); anti-GAPDH(60004-1-Ig, Proteintech, 1:1000) and anti-Calnexin (2679 T, Cell Signaling Technology, 1:1000).

### H&E staining

The iWAT, eWAT, BAT, and muscles of mice were permeated in 4% cold paraformaldehyde, soaked with paraffin, and next sectioned at 4 μm to determine the adipocytes of the cross-sectional area (CSA). The morphology of the pathological section was pictured by a microscope (Leica DMI 3000B microscope, Wetzlar, Germany).

### Enzyme-linked immunosorbent assay (ELISA)

The relative levels of IL-8 expression in the mouse serum and supernatant of cells were measured by the Mouse Interleukin 8 ELISA kit (Bio-Swamp, MU30010, China) following the manufacturer’s instructions.

### Statistical analysis

All statistical results were presented as mean ± SEM by SPSS Statistics 22.0 (IBM SPSS, Chicago), GraphPad Prism 8.0.1. All data of in vitro and animals were correspondingly calculated by the *Student’s T-test, One-way analysis of variance (ANOVA)* according to *Tukey’s* post hoc *tests*. Three independent experiments were performed to obtain high power of statistical data. The significance value deemed at *P < 0.05, **P < 0.01 and ***P < 0.001.

## Results

### Adipose wasting is observed in lung cancer and CRC cachexia mouse models

**Fig. 1:**
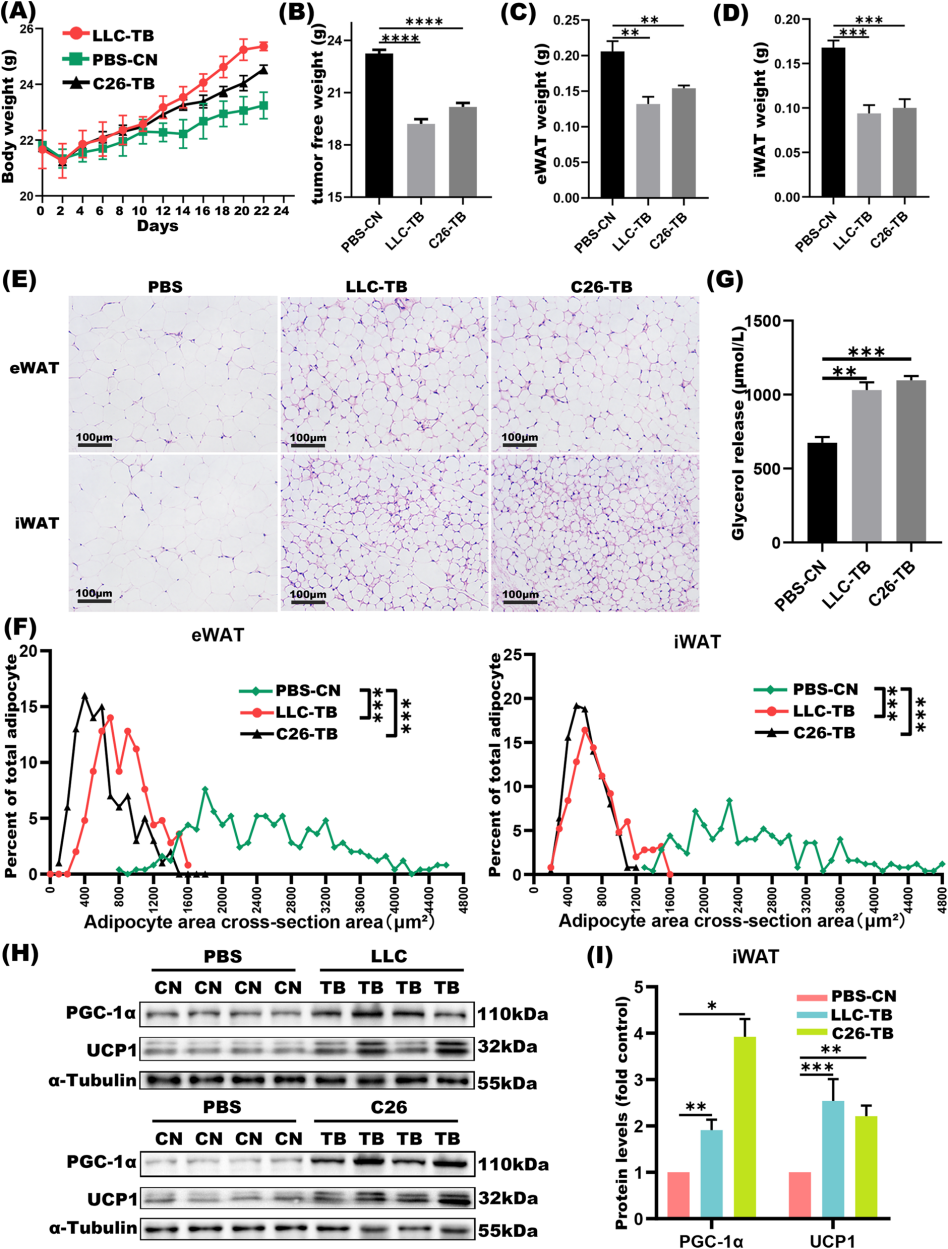
Adipocytes wasting is observed in mouse model of lung cancer and CRC cachexia. **A-D** The body weights growth curves of mice for 22 days in the LLC-TB, C26-TB and CN groups. Weight of the tumor free body was also assessed for mice (**B**), eWAT (**C**) and iWAT (**D**) in the CN and TB groups (*n* = 5), respectively. **E-F** H&E staining of eWAT, iWAT and the CSA of adipocytes in adipose tissues were analyzed by *χ2* analysis (*n* = 5). **G** Levels of glycerol release in mouse serum in the above groups. **H-I** Western blot of PGC1α and UCP1 proteins in the iWAT of CN and LLC-TB groups with densitometric analyses. *P < 0.05, **P < 0.01, ***P < 0.001, ****P < 0.0001

To explore the mechanism of adipocyte wasting driven by cancer-associated cachexia, we established the CC model by subcutaneously implanting LLC and C26 tumors, respectively. The body weights of mice in the TB groups increased in comparison to the CN group (Fig. 1A). However, the tumor-free body weight (Fig. 1B), in terms of the weights of iWAT, eWAT and GA muscles were lower in the TB groups (Fig. 1C-D, Fig. S1A). Adipocytes in the iWAT and eWAT tissues became more multilocular and smaller in the TB groups (Fig. 1E-F); together with higher level of glycerol release in the serum (Fig. 1G). Furthermore, Western blot revealed that the expression of the lipolysis markers, PGC1α and UCP1, in both iWAT and eWAT. Specifically, UCP1 in iWAT were increased in both LLC-TB and C26-TB group over their CN group (Fig. 1H-I); and PGC1α and UCP1 in the eWAT were similarly higher in the LLC-TB and C26-TB group than that in the controls (Fig. S1B-C). Adipose tissue wasting is often associated with muscle atrophy in CC [28]; indeed, relative fiber CSA of TA in the TB groups was lower than that in the CN group (Fig. S1D). These findings were consistent with that adipose wasting concurred with LLC and C26-induced cancer cachexia.

### Identification of IL-8 in EVs derived from LLC and C26 cells

**Fig. 2:**
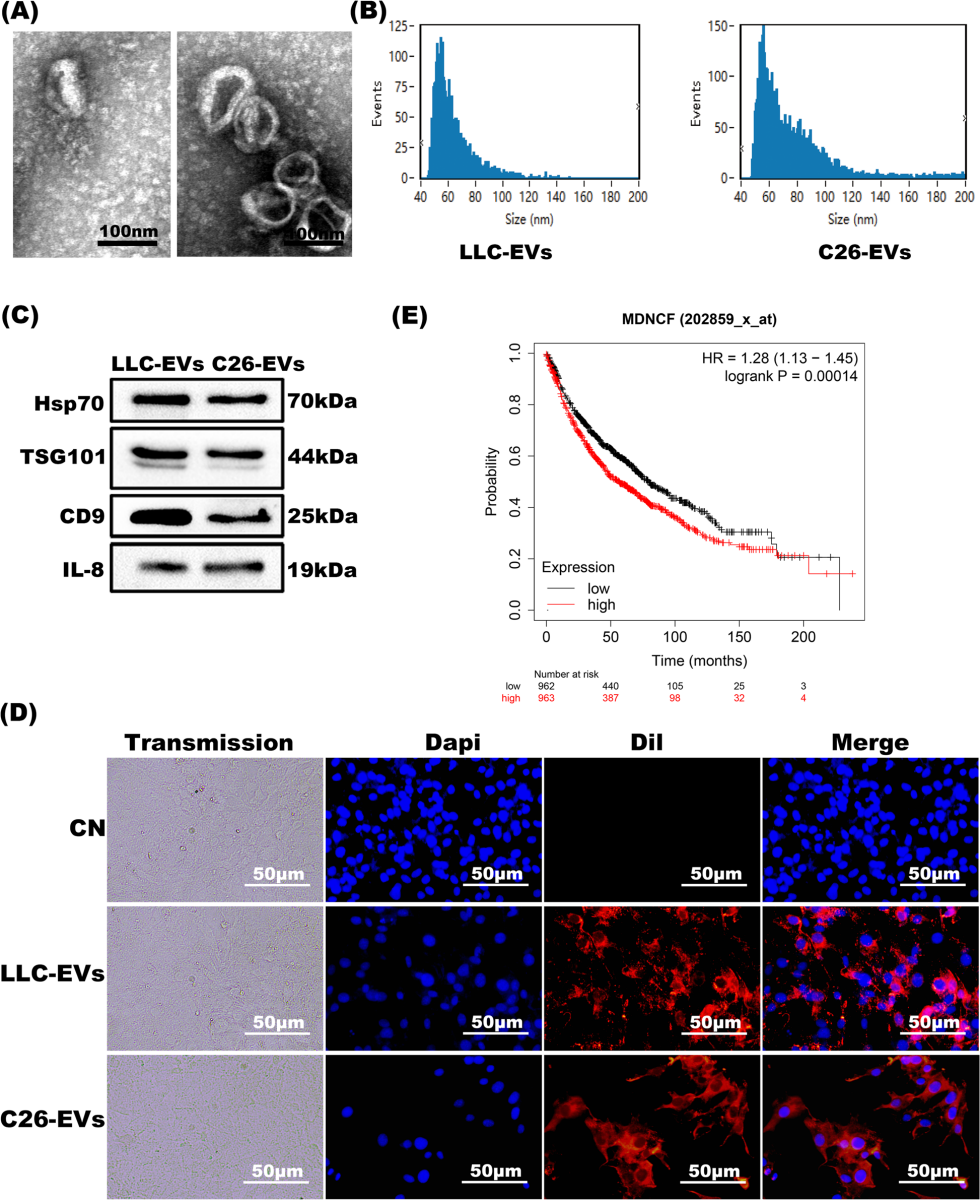
Identification of EVs isolated from LLC and C26 cells. **A** Morphological analysis of EVs derived from LLC and C26 cells by electron microscopy (bars = 100 nm). **B** Evaluating the particle size of EVs based on Nanoparticle Tracking Analysis (NTA). **C** Detection of EVs markers and IL-8 by Western blot. **D** 3 T3-L1 adipocytes were co-cultured with Dil-labeled LLC-EVs and C26-EVs (red) and fixed for confocal imaging. Scale bar =50 μm. **E** Kaplan–Meier analyses indicating reverse correlation between IL-8 expression in tumor tissues and overall survival of lung cancer cohorts

The EVs of LLC and C26 cells were collected from the culture medium supernatant. The morphology of EVs was analyzed under transmission electron microscopy (JEM 1200EX) and the average diameter of EVs was 50-200 nm (Fig. 2A-B). Western blot analyses showed IL-8 was enclosed in EVs from LLC and C26 cells (Fig. 2C). In comparison to 53.81 pg/mL of IL-8 in the CN group, its levels in mice serum in the LLC-TB and C26-TB groups were 383.7083 pg/mL and 218.6717 pg/mL, respectively. Furthermore, EVs marker proteins were present in EVs rather than in cell lysates (Fig. 2C, Fig. S2A). Additionally, internalizing of Dil-labeled LLC-EVs and C26-EVs were observed in 3 T3-L1 adipocytes for 24 hours (Fig. 2D).

IL-8 was known to increase in CRC over that in normal epithelial cells [29]; indeed, IL-8 expression was higher in the tumor and serum of patients with cachexia than in weight-stable patients [30]. The clinical data were next plotted with a KM-plot curve, dividing the patients into low- and high- expression groups based on IL-8 transcript expression in the tumor specimens obtained from lung cancer biopsies. Of importance, the overall survival was significantly lower in lung cancer patients expressing IL-8 mRNA in comparison to the median (HR = 1.28, log-rank *P* = 0.00014) (Fig. 2E).

### LLC and C26 cells derived-EVs are involved in 3 T3-L1 adipocytes wasting

**Fig. 3:**
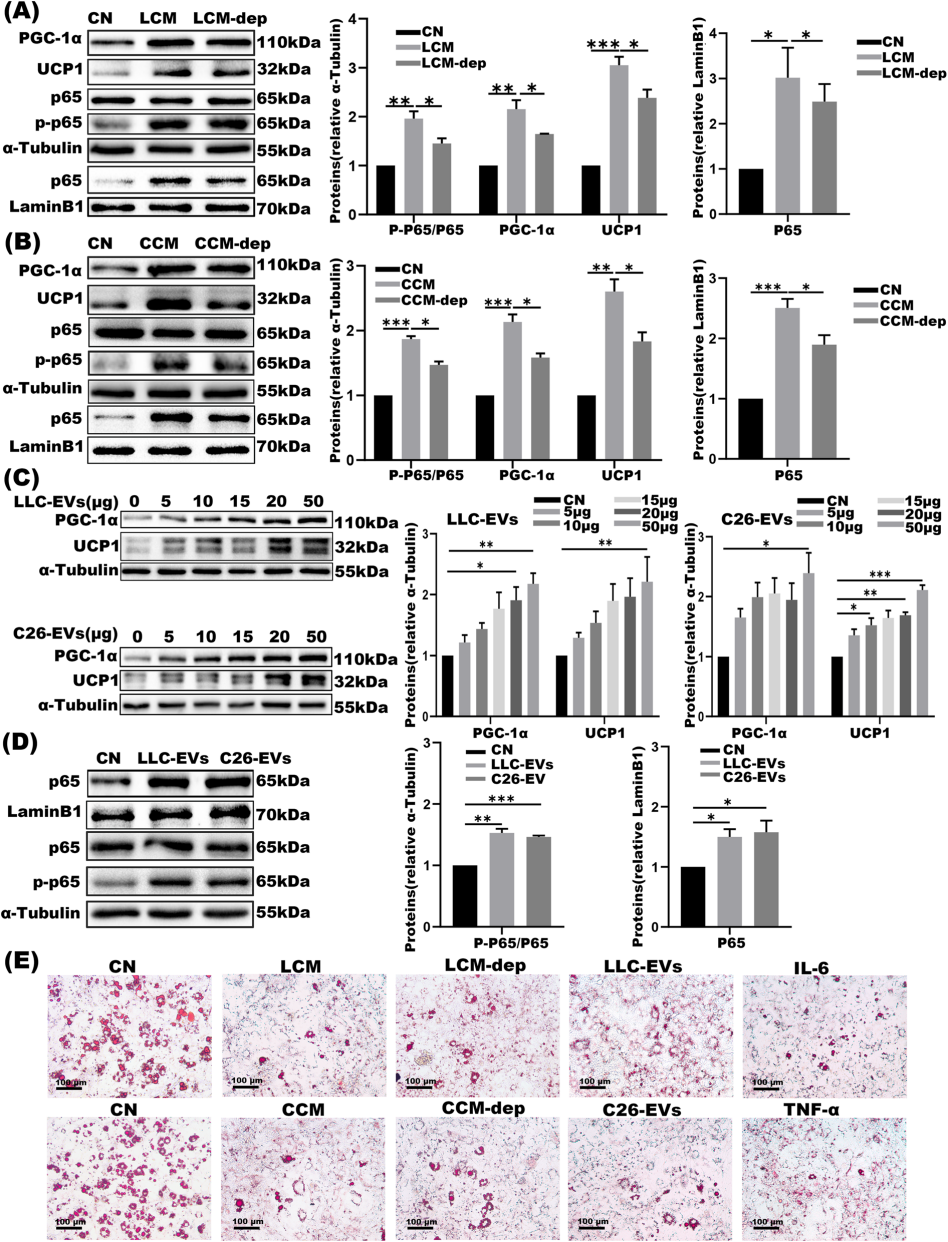
LLC and C26-derived EVs conferred lipolysis of 3 T3-L1 adipocytes. **A-B** Western blot and the densitometric analysis of UCP1, PGC1α and p-p65, total p65 protein expression in total extracts and nuclear extracts of 3 T3-L1 adipocytes, co-cultured with LLC versus C26-conditioned medium and EVs-free conditioned medium. **C-D** Western blot of PGC-1α and UCP1 in 3 T3-L1 adipocytes treated with LLC-EVs versus C26-EVs at various doses and the corresponding densitometric analyses (*n* = 3). **E** Oil red O staining of 3 T3-L1 adipocytes co-cultured with LCM, CCM, LCM-dep, CCM-dep, LLC-EVs (10 μg), C26-EVs (10 μg), IL-6 (50 ng/ml) and TNF-α (50 ng/ml). Bar = 100 μm. *P < 0.05, **P < 0.01,***P < 0.001

To assess the effects of tumor-derived EVs in CC adipocytes lipolysis, LLC/C26 cancer cell-conditioned medium (LCM and CCM) versus LCM/CCM depleted of EVs (LCM-dep and CCM-dep) were prepared as described [29, 31]. As shown, LCM and CCM-derived EVs evoked PGC1α and UCP1 expression together with p65 phosphorylation and nuclear translocation in 3 T3-L1 adipocytes; whereas this effect was less pronounced under LCM-dep/CCM-dep treatments (Fig. 3A-B). For further validation, 3 T3-L1 adipocytes were co-cultured with different dosage of LLC/C26-EVs. The outcomes demonstrated that activated p65, PGC1α, and UCP1 were all increased dose-dependently in association with lipolysis (Fig. 3C-E). These findings mechanistically linked EVs to adipocytes lipolysis.

### 3 T3-L1 adipocytes lipolysis are partially attributed to extracellular IL-8 level

**Fig. 4:**
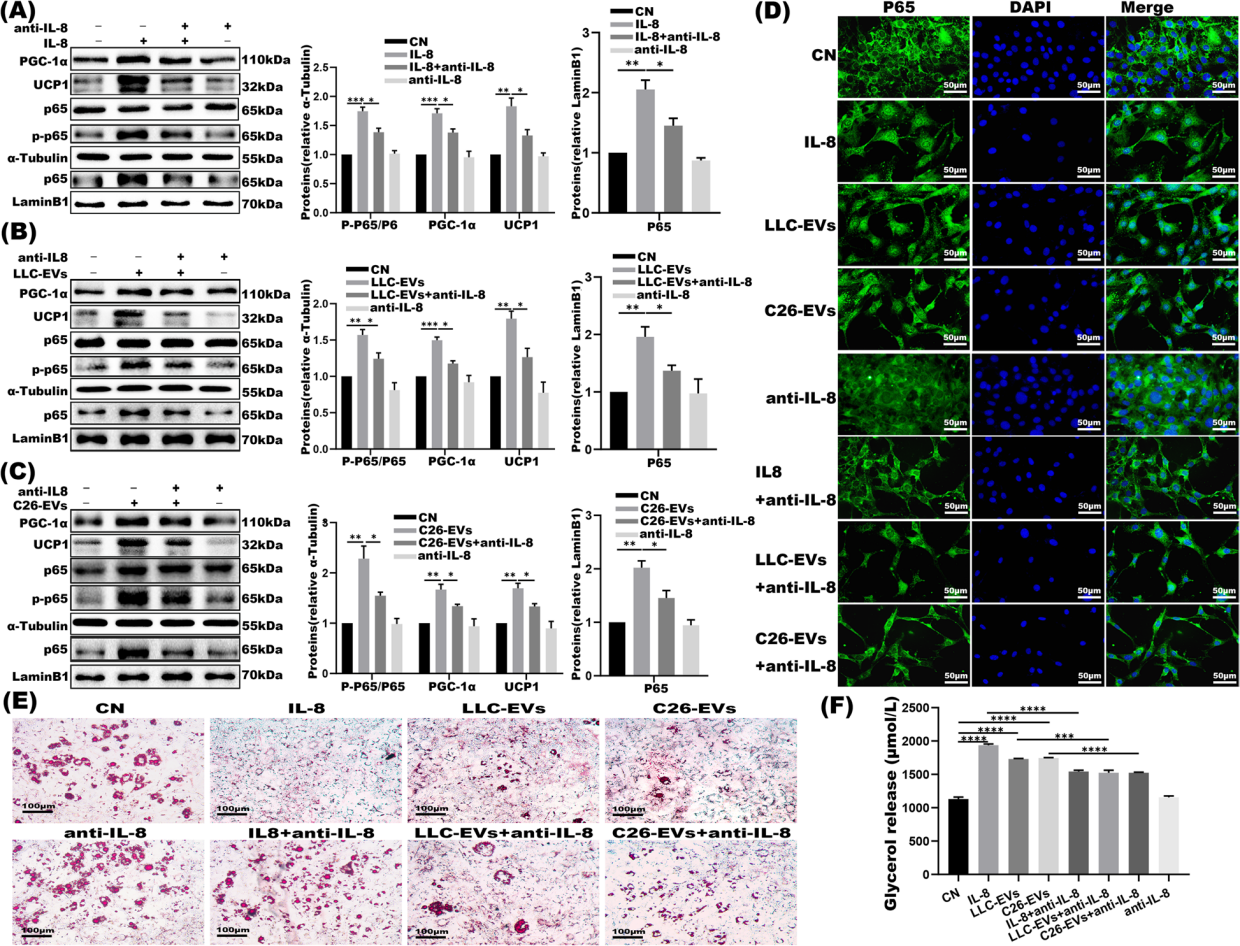
IL-8 in EVs induces lipolysis partly by activating NF-κB signaling pathway. **A-C** Western blot and the densitometric analysis of phosphorylated-p65, nuclear p65, total p65, UCP1, PGC1α in 3 T3-L1 adipocyte treated with IL-8, LLC-EVs (10 μg), C26-EVs (10 μg) and/or anti-IL-8 (*n* = 3), as indicated. **D** The activation of p65 NF-κB (as marked in Green) in 3 T3-L1 adipocytes was confocally imaged upon co-culturing with IL-8 (with and without specific anti-IL-8), LLC-EVs and/or C26-EVs (with and without specific anti-IL-8). The nucleus (Blue) was labeled with DAPI and 10 μg EVs were applied in test. **E** Oil red O staining assay for above groups. Bar = 100 μm. **F** Relative levels of glycerol release in cell supernatants upon above treatments. *P < 0.05, **P < 0.01, ***P < 0.001

To investigate whether an increase in IL-8 level was enough to induce adipocyte wasting, adipocytes were cultivated with recombinant IL-8 protein (rIL-8), LLC/C26-EVs, and/or neutralizing IL-8 antibody (anti-IL-8). Our study first demonstrated that p65 phosphorylation was markedly activated upon 40 min co-culture of mature 3 T3-L1 adipocytes and 10 ng/mL rIL-8. The mature 3 T3-L1 adipocytes were next co-cultured with rIL-8, LLC/C26-EVs and anti-IL-8.Specifically, PGC1α and UCP1 expression and the ratio of phosphorylated-p65/p65 were all increased upon rIL-8 and LLC/C26 EVs treatments; the effects were partly blunted by anti-IL-8 (Fig. 4A-C). Consistently, confocal imaging showed that both rIL-8 and EVs increased p65 nucleus translocation, an effect that largely disappeared upon anti-IL-8 treatment (Fig. 4D). In addition**,** Oil red O staining of adipocytes exposed substantially higher content of glycerol release in the supernatants upon rIL-8 and LLC/C26-EVs challenges and similarly, this effect was neutralized by anti-IL-8 (Fig. 4E-F).

### Adipocytes wasting is partially relieved by blocking IL-8/CXCR2 expression

**Fig. 5:**
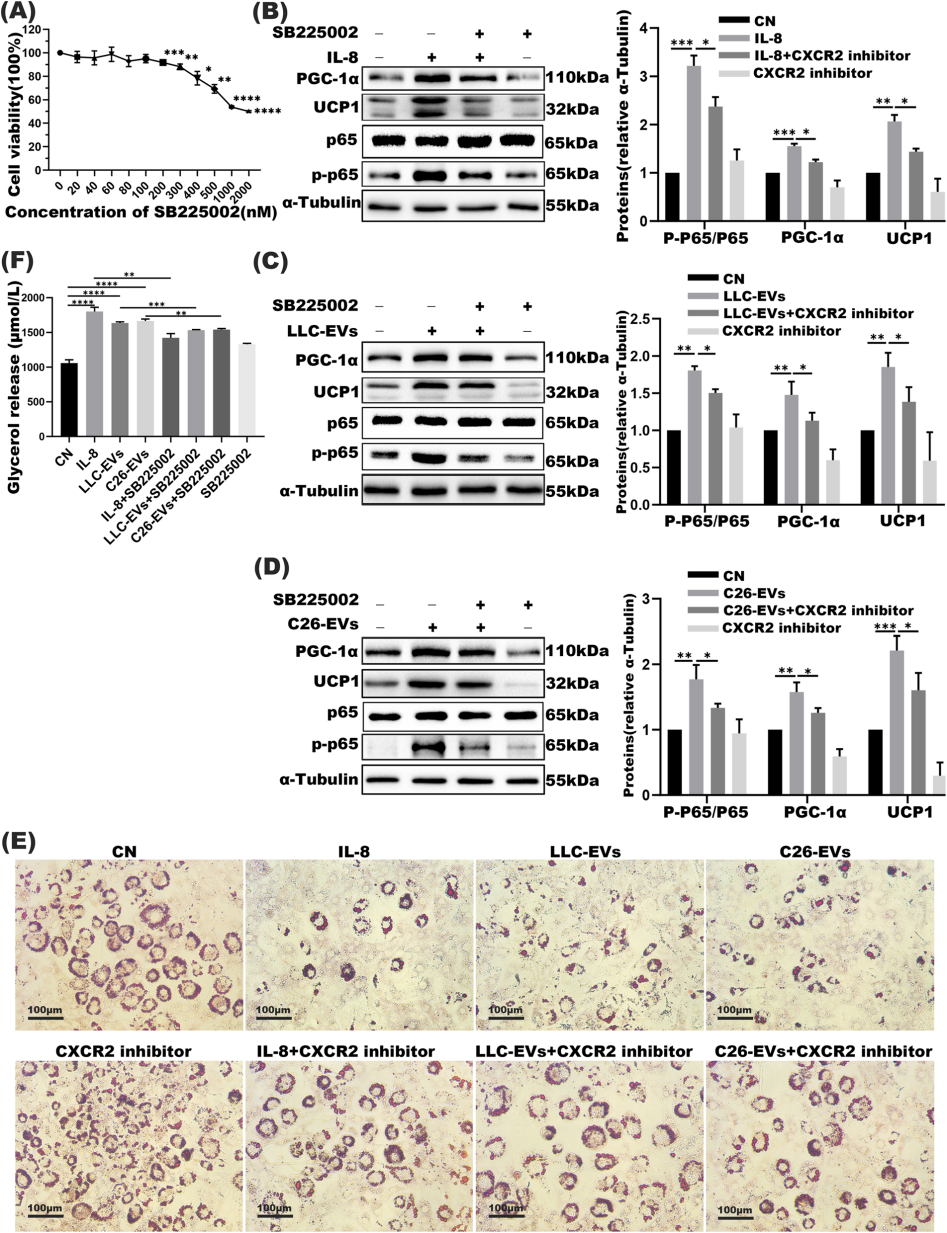
CXCR2 inhibitors partially relieved adipocyte wasting caused by IL-8 and EVs. **A** The viability of 3 T3-L1 adipocytes treated with different doses of CXCR2 inhibitor SB225002 as assessed by CCK8. **B-D** Western blot analyses of phosphorylated-p65, total p65, UCP1, and PGC1α in adipocytes co-cultured with IL-8 (with and without SB225002), LLC-EVs and/or C26-EVs (with and without SB225002) (*n* = 3), as indicated. 10 μg EVs were used in this experiment. **E** Oil red O staining test of the above groups. Bar =100 μm. **F** Relative levels of glycerol release in cell supernatants with the above treatment. 10 μg EVs were used in this experiment. *P < 0.05, **P < 0.01, ***P < 0.001

Muscle atrophy would be triggered by rIL-8 binding to its receptor CXCR2 rather than via smad3 or STAT3/5 signaling pathway [30]. In our study, we initiated the CXCR2 antagonist SB225002 test by monitoring its concentration and high dose (200 nM) was shown to bear marked cell cytotoxicity (Fig. 5A). Eventually, an intermediate concentration (50 nM) of SB225002 was elected for further experiments and western blot demonstrated rIL-8 and LLC/C26-EVs both motivated lipolysis in 3 T3-L1 mature adipocytes. Specifically, PGC1α and UCP1 expression in conjunction with the ratio of phosphorylated-p65/total p65 were all increased upon rIL-8 and LLC/C26-EVs treatment; while all these stimulatory effects were partially reversed upon SB225002 treatment (Fig. 5B-D). Outcomes of Oil red O staining assay were also consistent with increased glycerol release in the supernatants of adipocytes upon rIL-8 and LLC/C26-EVs challenges; however, the effect was attenuated by CXCR2 antagonist (Fig. 5E-F). These observations all supported induction of lipolysis of adipocyte through the IL-8/CXCR2 signaling by tumor-derived EVs.

### Inhibition of NF-κB/PGC1α signaling pathway represses IL-8 induced adipose wasting

**Fig. 6:**
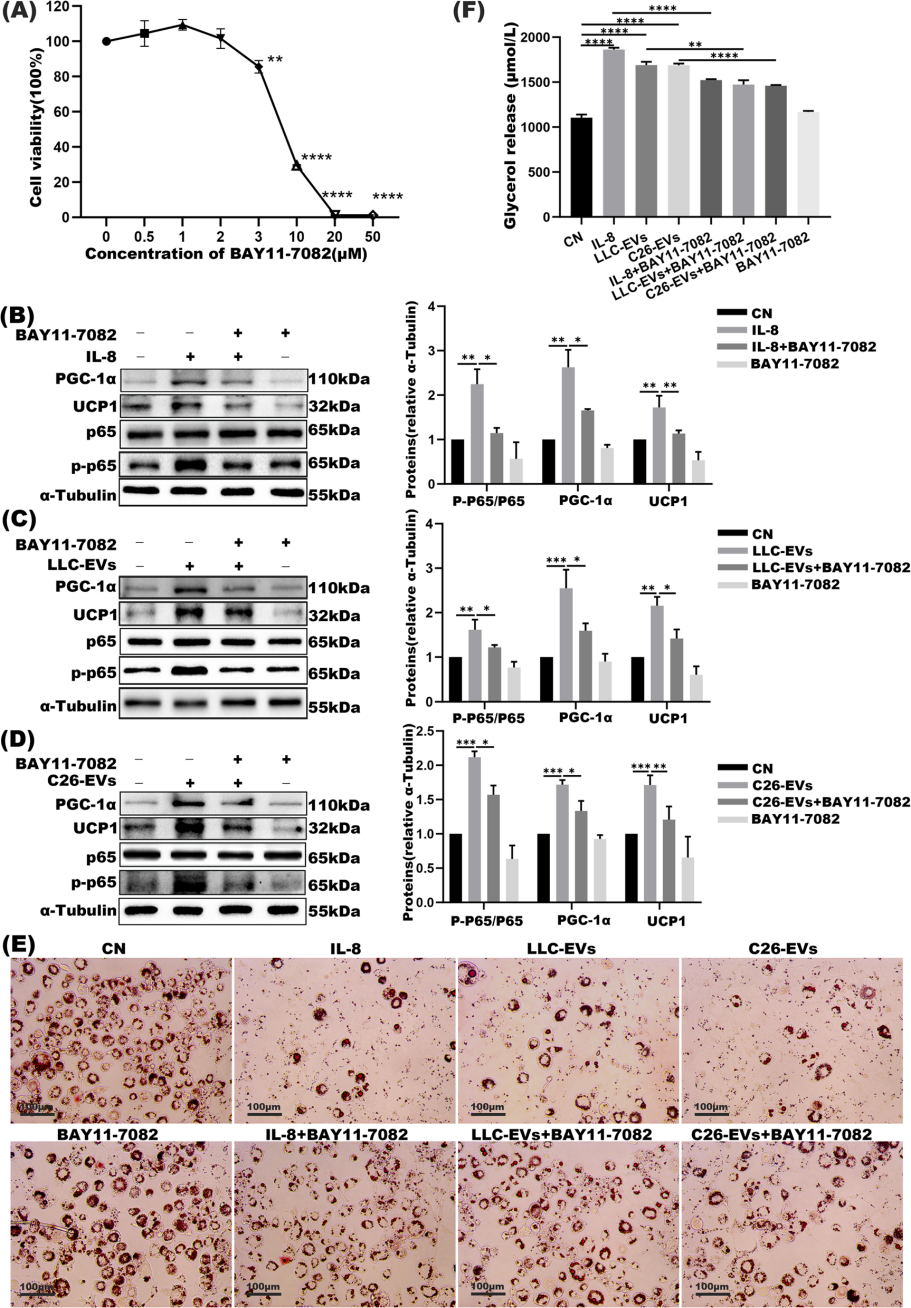
NF-κB inhibitors partially mitigated lipolysis induced by exosomal IL-8. **A** The viability of 3 T3-L1 adipocytes treated with different doses of NF-κB inhibitor BAY11-7082 as assessed by CCK8. **B-D** Western blot and the densitometric analysis of phosphorylated-p65, total p65, UCP1, and PGC1α in adipocytes co-cultured with IL-8 (with and without BAY11-7082), LLC-EVs and/or C26-EVs (with and without BAY11-7082). The blot shown were representatives of three independent experiments. 10 μg EVs were used in this experiment. **E** Oil red O staining was assessed in the above groups. Bar = 100 μm. **F** Relative levels of glycerol release in cell supernatants with the above treatments. *P < 0.05, **P < 0.01, ***P < 0.001

In pancreatic ductal adenocarcinoma, tumor-mediated SIRT1 loss was accounted for the induction of NF-κB signaling in cachectic muscles [32]. NF-κB pathway was similarly evoked in the liver, WAT, and skeletal muscle, triggering inflammation cascade in cancer cachexia via IL-1β, IL-6, and IL-8, etc. [20, 33]. Since IL-1β up-regulates the transcription of pro-inflammatory genes by stimulating NF-κB phosphorylation or inhibiting p65NF-κB methylation [34], we similarly assessed the activities of NF-κB pathway in the settings of adipose wasting in LLC and C26-derived cancer cachexia models. As shown, high dose (3 μM) of NF-κB inhibitor BAY11-7082 had pronounced cell cytotoxicity and would be avoided in subsequent tests; instead, an intermediate concentration (1 μM) was adopted to block the NF-κB signaling pathway (Fig. 6A). Mature 3 T3-L1 adipocytes were next co-cultured with rIL-8, LLC-EVs, C26-EVs and/or BAY11-7082. Adipocytes lipolysis was evaluated by Western blot, showing PGC1α and UCP1 expression and the ratio of phosphorylated-p65/total p65 increased upon rIL-8 and LLC/C26-EVs treatments and the enhancement was partially mitigated by BAY11-7082 (Fig. 6B-D). Similarly, both rIL-8 and EVs-mediated glycerol release were effectively blocked by BAY11-7082; and the reduction of lipid droplets in the adipocytes upon co-culture with rIL-8 and EVs was also markedly reversed by BAY11-708 (Fig. 6E-F).

## Discussion

High prevalence of cachexia is associated with lung, colorectal, pancreatic and gastric cancer patients who experienced muscle atrophy and adipocytes wasting, having a detrimental impact on patients’ quality of life [35, 36]. In this report, lung and colorectal cancer cachexia mouse model were established as previously described by using LLC and C26 cells [37]. The body weight of tumor-free mice and weight of adipose tissues were both lower in the LLC and C26-TB groups than in their control groups, respectively. Muscle mass loss and the cross sectional area of adipocytes in eWAT and iWAT were also decreased in the tumor groups (Fig. 1). These observations attested the successes in generating CC mouse models for subsequent tests.

Tumor cell-derived EVs bear multiple cargoes in the categories of proteins, lipids, and nucleic acid which via bloodstream would be further delivered to distant tissues and organs such as adipose tissues and muscles [38]. High expression of certain cytokines (including IL-6, IL-8, and TNF-α) was linked to cancer cachexia [39], as exemplified by that IL-6 enclosed in lung cancer-derived extracellular vesicles would evoke muscle wasting by activating STAT3 pathway [10, 40]. Elevation in IL-8 was also noted for its correlation with worse overall survival in lung cancer and CRC patient cohorts [41, 42]. Consistently, in advanced pancreatic cancer clinic cohorts, IL-8 expression was higher in cachectic patients in comparison to non-cachectic cases. Although negative correlation between IL-8 levels in the serum and muscle mass of pancreatic cancer patients was addressed [43], so far the function of IL-8 in adipose tissue was not reported yet. Our findings showed that IL-8 was expressed in both LLC and C26 EVs and IL-8 expression was higher in serum of the LLC and C26 cancer cachexia mice than their normal counterparts (Fig. 2). Analyses of clinical datasets indicated IL-8 expression in the patients with lung carcinoma was reversely associated with survival (Fig. 2E). Furthermore, tumor-derived EVs would be effectively internalized by 3 T3-L1 mature adipocytes to trigger downstream cascade. In our CC models, recombinant IL-8 stimulates adipocytes lipolysis; similarly, we previously found recombinant IL-6 mimicking TNFα to evoke the lipolysis of 3 T3-L1 adipocytes as well [10]. We determined that IL-8 neutralizing antibody could relieve 3 T3-L1 adipocytes lipolysis mediated by recombinant IL-8 protein and LLC/C26 EVs (Fig. 4), resembling LCM-dep and CCM-dep in which EVs were removed. Together, our observations evidenced that tumor-derived exosomal IL-8 actively contributed to lipolysis of adipocytes. In accordance, Phase I trial with neutralizing anti-IL-8 was undergoing in patients suffered from metastatic or un-resectable tumors [44].

IL-8-motivated NF-κB signaling is widely involved in the pathogenesis of hepatic, muscle diseases and cancer [45, 46]. The expression of p65NF-κB is significantly higher in the subcutaneous adipose tissue of patients with cachectic than in the control cohort [21, 22], indeed, our report was focused on assaying inflammation that has known relevance in CC adipocyte wasting. Our analyses systematically spanned typical markers including the expression of PGC1α and UCP1, the phosphorylation of p65NF-κB, and glycerol release. Taking complementary approaches, we parallelly examined LCM/EVs, IL-8, anti-IL-8, and CXCR2 and NF-κB inhibitors. Together, this study delineated the mechanisms of exogenous IL-8 in relevance to adipocytes lipolysis.

### Comparisons with other studies and what does the current work add to the existing knowledge

It was conceived that tumor-cell derived EVs played an active role in promoting adipocytes wasting in cancer cachexia [8]. Similarly, exosomal IL-6 and PTHrP were reported to induce adipocytes lipolysis [10, 47]. In pancreatic cancer, tumor-associated stromal cells released IL-8 to evoke muscle weight loss via the CXCR2-ERK1/2 cascade [43]. However, so far the relevance of EV-derived IL-8 has not been defined in CC adipocytes wasting. Our current objectives were designated to clarify the impacts of tumor-derived exosomal IL-8 in adipocytes lipolysis and mediating pathways, aimed to obtain novel therapeutic strategies against adipocytes wasting.

### Study strength and limitations

Our study positions exosomal IL-8 as an active mediator of CC adipocytes wasting. IL-8 expression and relevant mediating pathways were assessed in the serum of lung cancer and colorectal cancer mice models, together with clinical lung cancer cohorts. By pinpointing IL-8 and its downstream signaling in adipocytes lipolysis, our report offers potential therapeutic targets for CC adipocytes wasting intervention. The limitation is that it is not initiated on global and genomic methodologies, and thus, bears the bias on IL-8 selection and its established biological functions.

## Conclusions

Given the complexity of cachexia syndrome, there is no effective clinical intervention for cancer cachexia. This study showed that EVs derived from LLC and C26 tumor cells induced lipolysis in adipocytes, partially mediated by exosomal IL-8 and its downstream NF-κB signaling. We validated these findings with neutralizing IL-8 antibody and CXCR2 and NF-κB antagonists. In clinical settings, we affirmed IL-8 overexpression in tumor specimens datasets obtained from lung cancer biopsies. Together, we affirmed the correlations between IL-8 availability and unfavorable overall survival in lung cancer patients and adipocytes lipolysis in tumor-elicited pre-clinical models. By singling out IL-8 in the EVs cargos, we verified the relevance of targeting IL-8 and established pathways in lipolysis, positioning IL-8 as an important mediator of CC adipocytes wasting.

## Supplementary Information

**Additional file 1: Supplementary Fig. S1.** Muscle wasting was observed in cancer cachexia mice model. **A **The weight of the GA between CN and TB groups (*n* = 5). **B-C** Western blot analyses of PGC1α and UCP1 in eWAT of the TB and CN groups as indicated. **D** H&E staining of the TA and GA, respectively. Scale bar = 100 μm. *P < 0.05, **P < 0.01, ***P < 0.001

**Additional file 2: Supplementary Fig. S2.** Identification of EVs isolated from LLC and C26 tumor cells. **A** Western blot analyses of EVs markers, HSP70, CD9 and TSG101, together with the positive and negative controls (GAPDH versus Calnexin), respectively.

## Data Availability

Data and materials of this study are available from the corresponding author upon reasonable request.

## Abbreviations

CC: Cancer cachexia
LLC: Lewis lung carcinoma
CRC: Colorectal cancer
EVs: Exosomes
CM: Conditioned medium
IL-8: Interleukin-8
NF-κB: Nuclear transcription factor kappa-B
iWAT: Inguinal white adipose tissue
eWAT: Epididymal white adipose tissue
PGC1α: Peroxisome proliferative activated receptor,gamma,coactivator1
UCP1: Un-coupling protein1(mitochondrial, protein carrier)
CXCR2: C-X-C motif chemokine receptor 2
LCM/CCM-dep: LLC/C26 cells-derived conditioned medium dispose EVs.
