# Supplementary material for: Exosomal IL-8 derived from Lung Cancer and Colon Cancer cells induced adipocyte atrophy via NF-κB signaling pathway
Source: Lipids Health Dis. 2022 Dec 29;21:147. doi: 10.1186/s12944-022-01755-2 (PMC9798689; doi:10.1186/s12944-022-01755-2)

Supplementary Figure.S1. Muscle wasting was observed in cancer cachexia mice model. (A) The weight of the GA between CN and TB groups (n=5). (B-C) Western blot analyses of PGC1α and UCP1 in eWAT of the TB and CN groups as indicated. (D) H&E staining of the TA and GA, respectively. Scale bar=100 μm. **P*<0.05, ***P*<0.01, ****P*<0.001.


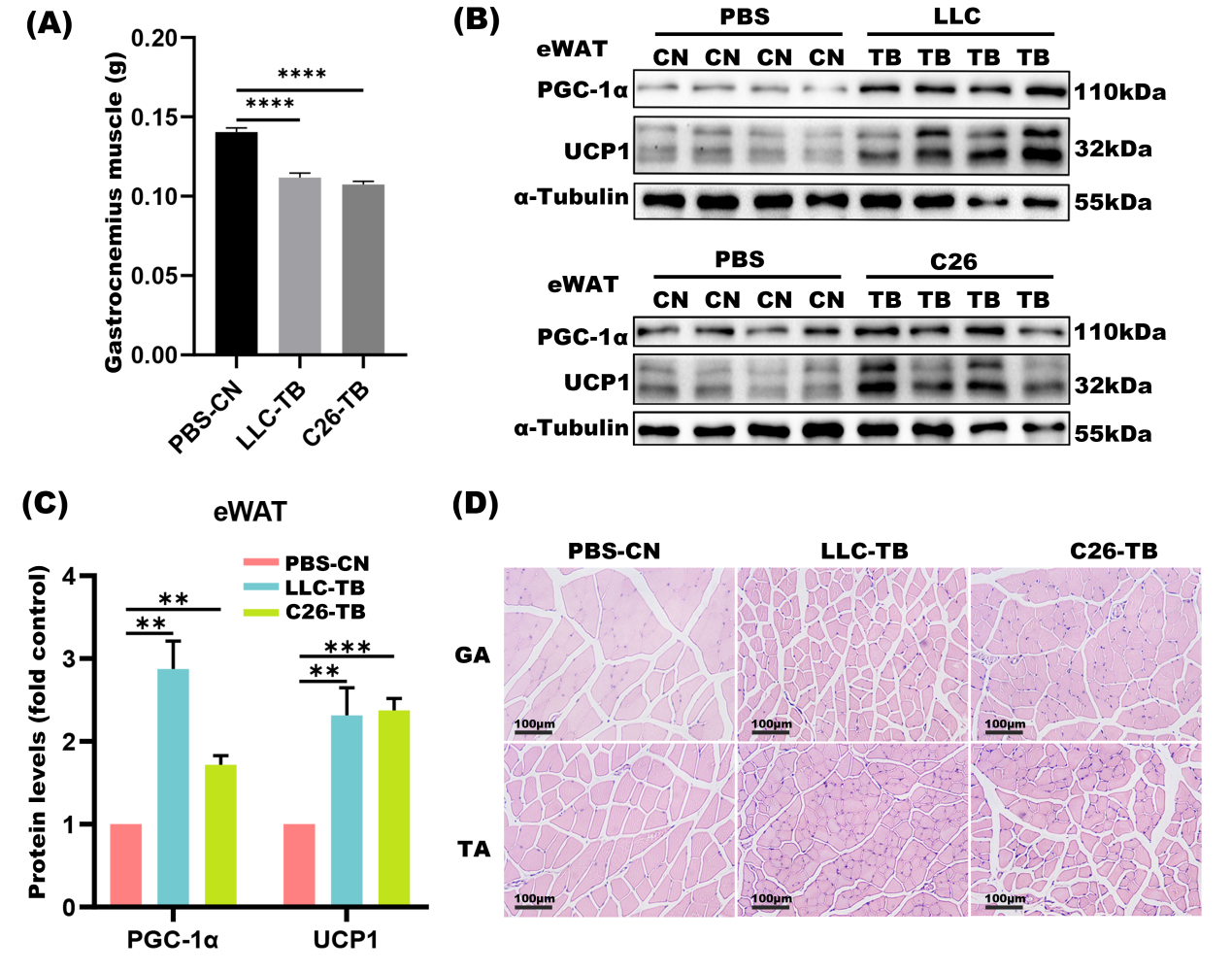

Supplement: Supplementary file 1 — Additional file 1: Supplementary Fig. S1. Muscle wasting was observed in cancer cachexia mice model. A The weight of the GA between CN and TB groups (n = 5). B-C Western blot analyses of PGC1α and UCP1 in eWAT of the TB and CN groups as indicated. D H&E staining of the TA and GA, respectively. Scale bar = 100 μm. *P < 0.05, **P < 0.01, ***P < 0.001 [file 12944_2022_1755_MOESM1_ESM.docx]
