# Supplementary material for: Exosomal IL-8 derived from Lung Cancer and Colon Cancer cells induced adipocyte atrophy via NF-κB signaling pathway
Source: Lipids Health Dis. 2022 Dec 29;21:147. doi: 10.1186/s12944-022-01755-2 (PMC9798689; doi:10.1186/s12944-022-01755-2)

Supplementary Figure.S2. **Identification of EVs isolated from LLC and C26 tumor cells**. (**A**) Western blot analyses of EVs markers, HSP70, CD9 and TSG101, together with the positive and negative controls (GAPDH versus Calnexin), respectively.


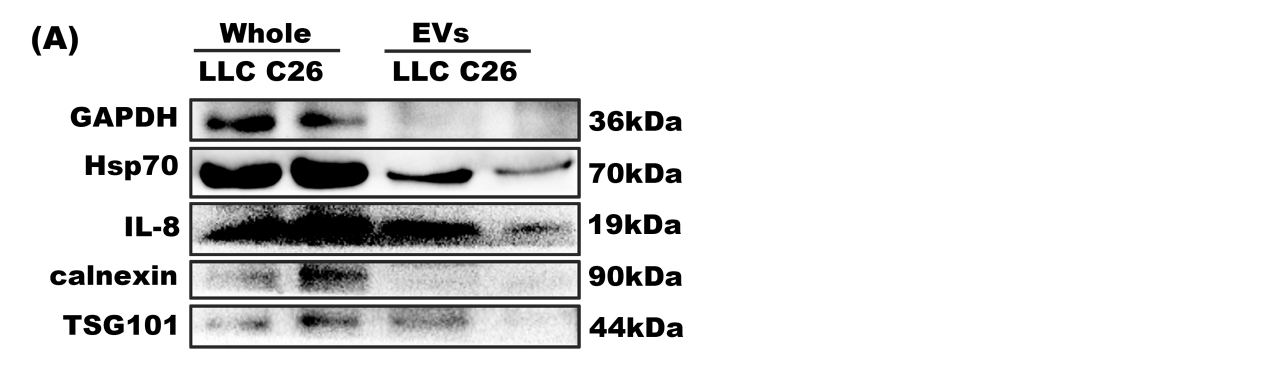

Supplement: Supplementary file 2 — Additional file 2: Supplementary Fig. S2. Identification of EVs isolated from LLC and C26 tumor cells. A Western blot analyses of EVs markers, HSP70, CD9 and TSG101, together with the positive and negative controls (GAPDH versus Calnexin), respectively. [file 12944_2022_1755_MOESM2_ESM.docx]
